# An Extragastrointestinal Stromal Tumor Arising From the Omentum in a Young Hispanic Male

**DOI:** 10.7759/cureus.58824

**Published:** 2024-04-23

**Authors:** Nathan DeRon, Huda N Khan

**Affiliations:** 1 Internal Medicine, Methodist Health System, Dallas, USA

**Keywords:** egist, tyrosine kinase inhibitor, cd117, kit, extragastrointestinal, gastrointestinal stromal tumor, gist

## Abstract

Gastrointestinal stromal tumors (GISTs) are neoplasms arising from the bowel wall, most often in the jejunoileum of the small intestine, but rarely from extragastrointestinal locations. GISTs most often occur in patients older than 40 years of age and can present with a multitude of gastrointestinal symptoms. We present a rare case of an extragastrointestinal stromal tumor (EGIST) causing abdominal pain and melena in a 34-year-old Hispanic male. The patient presented with diffuse abdominal pain, melena, and severe anemia. Computed tomography of the abdomen revealed a large mass abutting the small bowel. The patient was taken to surgery where the mass, which appeared to be deriving from the omentum and invading the adjacent small bowel, was completely excised and found to be a spindle cell GIST. Excision margins were determined to be negative, and the patient was started on a tyrosine kinase inhibitor for maintenance therapy. The patient continues to follow up on an outpatient basis for surveillance. This case represents the rare disease entity EGIST presenting outside the typical demographics of the disease in a young patient with no identified previous genetic syndromes. Gross examination of the mass in this case was also atypical given the appearance that the mass was rooted in the omentum and invading the small bowel which would suggest the primary tumor site was extragastrointestinal. This case demonstrates the need to build a differential diagnosis that includes GIST and the ability to successfully treat this disease if it is identified early in the clinical course.

## Introduction

Gastrointestinal stromal tumors (GISTs) are neoplasms arising from the bowel wall, most often in the jejunoileum of the small intestine, but rarely from extragastrointestinal locations [1]. Extragastrointestinal locations include the soft tissues of the abdomen such as the peritoneum, mesentery, and omentum [1,2]. These primary extragastrointestinal GISTs comprise fewer than 5% of cases relative to GISTs arising from the gastrointestinal (GI) tract [2-4].

GISTs typically arise in older adults with a median age of diagnosis of approximately 65 years of age, but 10% occur in patients younger than 40 years of age [5,6]. There are vast differences in incidence between races as well, with the majority of cases occurring in African Americans, Caucasians, and Asian or Pacific Islanders [7]. GISTs are also associated with genetic syndromes such as neurofibromatosis type 1, Carney-Stratakis syndrome, and Carney triad [8].

We present a rare case of an extragastrointestinal stromal tumor (EGIST) in a 34-year-old Hispanic male and describe the diagnostic approach and therapeutic techniques applied to the case.

## Case presentation

A 34-year-old Hispanic male with no significant past medical history presented to the emergency department with three days of diffuse abdominal pain, nausea, and black and maroon-colored tarry stools which had become progressively loose. The patient denied any bright red blood per rectum or hematemesis. Additional history revealed no obvious risk factors for GI hemorrhage such as heavy use of non-steroid anti-inflammatory medications, alcohol use, or history of acid reflux disease. The patient denied any recent medication changes and a history of tobacco and illicit drug use but did report occasional alcohol use. The patient denied any history of abdominal surgeries.

The patient did report a social history positive for anal-receptive intercourse with his male partner and reported his last receptive coitus was increasingly painful. Further information from the patient’s partner revealed a recent history of the patient becoming increasingly pale, light-headed, and feeling unsteady when standing without any episodes of syncope or loss of consciousness.

**Figure 1:**
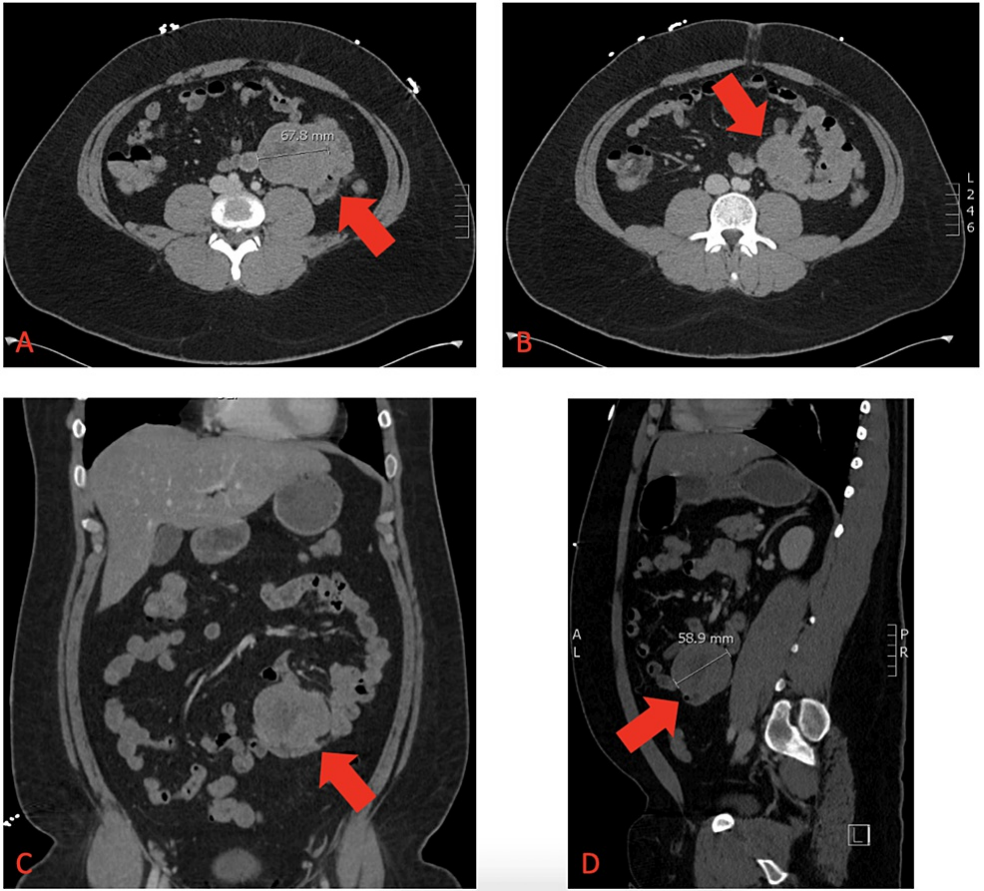
Left mesenteric mass measuring 8.7 x 6.5 x 5.5 cm. (A) Axial CT view illustrating left mesenteric mass abutting adjacent the small bowel. (B) Axial CT view with redemonstration of the mass with local small bowel. (C) Coronal CT view of the left abdominal mass arising from the omentum and involving local small bowel loops. (D) Sagittal CT view of the abdominal mass showing proximity to local structures. CT = computed tomography

Initial vital signs and laboratory data were pertinent for tachycardia with a heart rate between 120 and 130 beats per minute, systolic blood pressure of approximately 100 mmHg, hemoglobin of 5.6 g/dL, white blood cell count of 17,100/µL, lactate of 3.4 mmol/L, and blood urea nitrogen of 28 mg/dL. The physical examination revealed severe, diffuse abdominal pain to light palpation without significant abdominal distention. Bowel sounds were active. A computed tomography (CT) study of the patient’s abdomen and pelvis with intravenous contrast was ordered upon initial presentation for further evaluation of a potential source of GI bleeding. The CT revealed a 6 x 7 x 5 cm mass in the mesentery of the left abdomen abutting multiple loops of the small bowel without dilation (Figure 1). There was no evidence of any potential metastatic disease on the imaging study.

The patient was immediately started on intravenous proton pump inhibitor and octreotide infusions and provided multiple transfusions of packed red blood cells. The GI, general surgery, and interventional radiology (IR) services were consulted. Esophagogastroduodenoscopy was unrevealing, and IR recommended surgical intervention rather than biopsy given the high-risk location of the mass.

The patient was taken to the operating room for an exploratory laparoscopy with general surgery. The mesenteric mass was encountered at the mid-jejunum, and the procedure was converted to laparotomy. It appeared to be rooted in the omentum and invading the adjacent small bowel. There was no evidence of bowel obstruction or perforation. The mass was resected, and an intraoperative consultation with pathology was made. Frozen sections were derived, and the pathology evaluation revealed a spindle cell neoplasm favored to be a GIST. The tumor was fully resected with what appeared to be clean margins. Resection anastomosis of the adjacent small bowel was performed, and the patient’s abdomen was closed.

**Figure 2:**
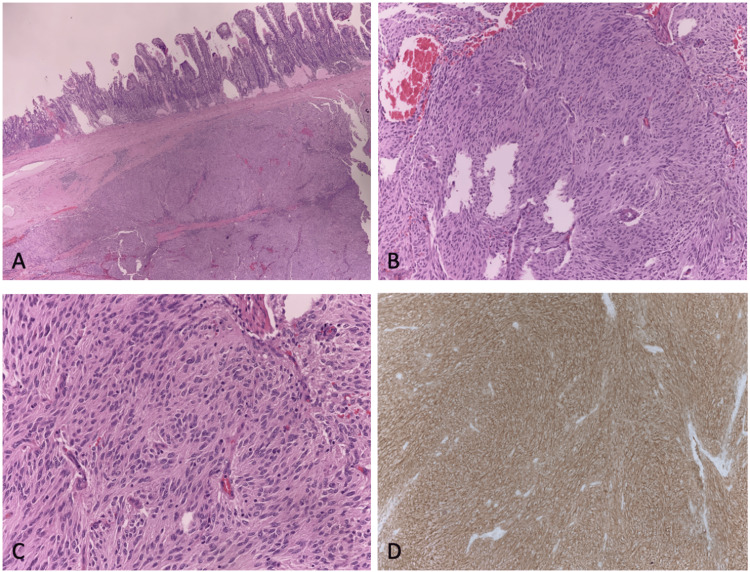
Pathology slides of the GIST in this case. (A) Low-magnification image with H&E stain showing invasion of the small bowel. (B) Medium-magnitude H&E stain image of tumor body showing high mitotic activity. (C) High-magnitude image with H&E stain showing thorough invasion of neoplastic spindle cells. (D) Image illustrating uptake of brown KIT stain indicating elevated KIT/CD117 activity. GIST = gastrointestinal stromal tumor; H&E = hematoxylin and eosin

The pathology report revealed a low-grade pT3, pN0 GIST measuring 8.7 x 6.5 x 5.5 cm. The mitotic rate was one per five square millimeters. Three local lymph nodes were resected without any indication of malignant cells. The margins of the tumor were confirmed to be negative. The tumor showed positive staining for KIT and negative staining for SMA, desmin, and CD34 (Figure 2).

The patient improved postoperatively with stable vitals and resolution of bloody stools. On oncology consultation, the patient was started on imatinib 400 mg daily. The patient underwent further lab testing which showed no indication of toxicity from immunotherapy. At the last outpatient follow-up, the patient was scheduled for restaging imaging and will continue to follow up with the oncology outpatient service for ongoing management and monitoring.

## Discussion

Establishing the diagnosis of GIST is challenging due to the rarity of the disease, especially in the younger population. The incidence of primary EGIST is less than 5%, and even rarer in younger individuals. The presentation of this disease often mimics other causes of GI bleeds such as diverticular hemorrhage, angiodysplasia, and colonic malignancy, implying that the differential is vast and encompasses myriad disease possibilities. The patient in this case presented with abdominal pain and melenic stools with occasional watery diarrhea, and even after imaging revealed the presence of an abdominal mass, both carcinoid malignancy and lymphoma were still higher on the differential than GIST.

Even rarer is that it grossly appeared to be rooted in the omentum with only secondary involvement with the adjacent small intestine. All GISTs are thought to originate from interstitial cells of Cajal which reside in the muscular layer of the bowel and appear to help regulate peristalsis [9]. EGISTs are thought to arise due to sites of interstitial cells of Cajal which were dispersed in the soft tissue of the abdomen during embryogenesis [10].

Although the most common primary sites are the stomach, jejunum, and ileum, one characteristic of GISTs is the ability to arise from any location throughout the entire GI tract. This characteristic makes diagnosing this disease extremely difficult as presenting symptoms can range from dysphagia and melena to obstructive jaundice and bowel obstruction [11]. Rectal tumors can present as hematochezia or even with primary urinary symptoms due to mass effect, especially in men when the tumor abuts the prostate [12]. The patient in this case presented with melena and profound anemia but no signs of bowel obstruction making it exceedingly difficult to quickly identify this disease process.

GISTs are known to be caused by mutations in the KIT, or CD117, oncogene causing uninhibited cell proliferation [13]. In some cases, mutations in the platelet-derived growth factor receptor alpha are also implicated in the neoplastic process [14]. Mutations in other genes causing GISTs are less frequent. The most common type of GIST is the spindle cell type which accounts for approximately 70% of all GISTs and exhibits uniform eosinophilic cells with spindling [15].

Initial workup for GISTs includes abdominal and pelvic imaging specifically using CT with intravenous and oral contrast to help define the bowel margins [16]. Magnetic resonance imaging and positron emission tomography may help define the mass but are less useful in this scenario. The diagnostic gold standard is biopsy with immunohistochemical staining and pathology review. Biopsy may be pursued using upper or lower endoscopy with endoscopic ultrasound guidance or with surgical intervention depending on location and tumor burden. As demonstrated, this case revealed a spindle cell phenotype and suggestion of GIST during the intraoperative pathology consultation encouraging the attempt to excise the entire tumor.

Initial therapeutic strategies for GISTs include surgical excision and pathologic evaluation with immunohistochemistry and molecular testing to ensure negative margins [17] which was accomplished in this case. During this time, it is exceedingly important to establish negative borders of the excised tissue as this will help establish the likelihood of recurrence.

After excision and surgical recovery, maintenance therapy of the neoplasm may be pursued. For tumors larger than 2 cm, chest imaging with X-ray or CT is recommended [16]. Given the association of GISTs with genetic syndromes, it is important to have younger patients evaluated for mutations as they may be at increased risk for further disease from these syndromes. Oncology consultation may also be helpful in these cases for the evaluation of adjuvant immunotherapy with a tyrosine kinase inhibitor as this therapy has been shown to reduce the likelihood of recurrence after three years of therapy [18].

## Conclusions

Given the rarity of GISTs and the array of presenting symptoms, the diagnosis is often overlooked. EGISTs are even rarer and may present late depending on the location and impact on presenting symptoms. The patient in this case was a Hispanic male under the age of 40 which is outside the typical demographic for GISTs. This case demonstrates the importance of building a differential that includes GIST as a potential diagnosis even in patients who do not fit the typical demographic for the disease. Diagnosis of GIST is also important given its ability to be effectively managed with positive outcomes. If patients are appropriately diagnosed in a timely manner, surgical intervention and adjuvant immunotherapy can be curative which would improve overall clinical outcomes and reduce both the morbidity and mortality of this disease.

## References

[1] Miettinen M, Lasota J (2001). Gastrointestinal stromal tumors--definition, clinical, histological, immunohistochemical, and molecular genetic features and differential diagnosis. Virchows Arch.

[2] Reith JD, Goldblum JR, Lyles RH, Weiss SW (2000). Extragastrointestinal (soft tissue) stromal tumors: an analysis of 48 cases with emphasis on histologic predictors of outcome. Mod Pathol.

[3] Kataoka M, Saitoh T, Kawashima K (2021). Primary extragastrointestinal stromal tumor of greater omentum with intraperitoneal bleeding. Intern Med.

[4] Akolkar S, Melitas C, Piper M (2019). Pelvic gastrointestinal stromal tumor with pulmonary metastasis. ACG Case Rep J.

[5] Søreide K, Sandvik OM, Søreide JA, Giljaca V, Jureckova A, Bulusu VR (2016). Global epidemiology of gastrointestinal stromal tumours (GIST): a systematic review of population-based cohort studies. Cancer Epidemiol.

[6] Nishida T, Blay JY, Hirota S, Kitagawa Y, Kang YK (2016). The standard diagnosis, treatment, and follow-up of gastrointestinal stromal tumors based on guidelines. Gastric Cancer.

[7] Ulanja MB, Rishi M, Beutler BD (2019). Racial disparity in incidence and survival for gastrointestinal stromal tumors (GISTs): an analysis of SEER database. J Racial Ethn Health Disparities.

[8] Recht HS, Fishman EK (2020). Carney-Stratakis syndrome: a dyad of familial paraganglioma and gastrointestinal stromal tumor. Radiol Case Rep.

[9] Foong D, Zhou J, Zarrouk A, Ho V, O'Connor MD (2020). Understanding the biology of human interstitial cells of Cajal in gastrointestinal motility. Int J Mol Sci.

[10] Franzini C, Alessandri L, Piscioli I (2008). Extra-gastrointestinal stromal tumor of the greater omentum: report of a case and review of the literature. World J Surg Oncol.

[11] Scola D, Bahoura L, Copelan A, Shirkhoda A, Sokhandon F (2017). Getting the GIST: a pictorial review of the various patterns of presentation of gastrointestinal stromal tumors on imaging. Abdom Radiol (NY).

[12] Parab TM, DeRogatis MJ, Boaz AM (2019). Gastrointestinal stromal tumors: a comprehensive review. J Gastrointest Oncol.

[13] Klug LR, Bannon AE, Javidi-Sharifi N (2019). LMTK3 is essential for oncogenic KIT expression in KIT-mutant GIST and melanoma. Oncogene.

[14] Grunewald S, Klug LR, Mühlenberg T (2021). Resistance to avapritinib in PDGFRA-driven GIST is caused by secondary mutations in the PDGFRA kinase domain. Cancer Discov.

[15] Wada R, Arai H, Kure S, Peng WX, Naito Z (2016). "Wild type" GIST: clinicopathological features and clinical practice. Pathol Int.

[16] Begum FA, Rahman MA, Rabbi H, Mostofa G, Chowdhury Q (2019). Primary jejunal gastrointestinal stromal tumor: diagnosis delay of 3 years but successful management in early stage (II) by surgery and adjuvant therapy. Gastrointest Tumors.

[17] Florin CM, Bogdan F, Cristian L, Maria TA, Mihai D, Viorel S (2020). Surgical treatment of gastric GIST: feasibility of laparoscopic resection and postoperative outcome. J Coll Physicians Surg Pak.

[18] von Mehren M, Joensuu H (2018). Gastrointestinal stromal tumors. J Clin Oncol.

